# Continental-scale migration patterns and origin of *Helicoverpa zea* (Lepidoptera: Noctuidae) based on a biogeochemical marker

**DOI:** 10.1093/ee/nvae034

**Published:** 2024-04-18

**Authors:** Silvana V Paula-Moraes, Eduardo S Calixto, Abraão A Santos, Francis P F Reay-Jones, Dominic D Reisig, Yasmine Farhan, Jocelyn L Smith, William D Hutchison

**Affiliations:** Entomology and Nematology Department, West Florida Research and Education Center, University of Florida, Jay, FL 32565, USA; Entomology and Nematology Department, West Florida Research and Education Center, University of Florida, Jay, FL 32565, USA; Entomology and Nematology Department, West Florida Research and Education Center, University of Florida, Jay, FL 32565, USA; Department of Plant and Environmental Sciences, Clemson University, Florence, SC 29501, USA; Department of Entomology and Plant Pathology, North Carolina State University, Raleigh, NC 27695, USA; Department of Plant Agriculture, University of Guelph, Ridgetown Campus, Ridgetown, ON, Canada; Department of Plant Agriculture, University of Guelph, Ridgetown Campus, Ridgetown, ON, Canada; Department of Entomology, University of Minnesota, St. Paul, MN 55108, USA

**Keywords:** corn earworm, hydrogen isotope, migration, lagarta da espiga, mariposas-praga, isótopos de hidrogênio, migração, Manejo Integrado de Pragas (MIP), Manejo da Resistência de Insetos (MRI)

## Abstract

Insect migrations have ecological and economic impacts, particularly in agriculture. However, there is limited knowledge about the migratory movements of pests at the continental scale, which is an important factor influencing the spread of resistance genes. Understanding the migratory patterns of economic pests, like *Helicoverpa zea* (Boddie), is essential for improving Integrated Pest Management (IPM) and Insect Resistance Management (IRM) strategies. In this study, we used stable hydrogen isotopic ratios in wing tissue as a biogeochemical marker to examine migratory patterns and estimate the native origins of *H. zea* individuals collected across a wide latitudinal gradient in North America. Samples collected at higher latitudes (Ontario, Canada and Minnesota, USA) exhibited a greater proportion (60%–96%) of nonlocal individuals, with an increased probability of origin from the southeastern United States. Populations from mid-latitudes (Florida, North Carolina, and South Carolina) showed a blend of local and nonlocal (40%–60%) individuals. Finally, 15% of the southernmost population individuals (Puerto Rico) were classified as migratory, with some having a probability of origin at higher latitudes (>30°). Overall, our results provide evidence of a northward spring/summer migration of *H. zea* in North America and underscore the significance of the southeastern United States as a hub for genetic flow. In addition, based on stable hydrogen isotopic ratios, there is strong evidence of reverse (southward) migration of *H. zea* from the continental United States to Puerto Rico. Our study highlights the implications for IPM and IRM programs and the need for management strategies that account for both northward and southward migration patterns.

## Resumo

Migrações de insetos têm impactos ecológicos e econômicos significativos, especialmente na agricultura. No entanto, existe um conhecimento limitado sobre os movimentos migratórios de pragas em escala continental, o que é um fator determinante na disseminação de genes de resistência. Entender os padrões migratórios de pragas econômicas, e.g., *Helicoverpa zea* (Boddie), é essencial para o aprimoramento das estratégias de Manejo Integrado de Pragas (MIP) e da Resistência de Insetos (MRI). Neste estudo, utilizamos isótopos estáveis de hidrogênio presentes nas asas das mariposas como marcador biogeoquímico para examinar os padrões migratórios e estimar a origem de indivíduos de *H. zea* coletados em um amplo gradiente latitudinal na América do Norte. Amostras coletadas em latitudes mais altas (Canadá e Minnesota, EUA) exibiram uma proporção maior (60-96%) de indivíduos não locais, com uma alta probabilidade de origem na região sudeste dos EUA. Populações de latitudes médias (Flórida, Carolina do Norte e Carolina do Sul) mostraram uma mistura de indivíduos locais e não locais (40-60%). Finalmente, 15% dos indivíduos da população mais ao sul (Porto Rico) foram classificados como migratórios, com alguns tendo alta probabilidade de origem em latitudes mais altas (> 30°). No geral, nossos resultados fornecem evidências de uma migração durante primavera/verão de *H. zea* para o norte na América do Norte, e também destacam a importância do sudeste dos EUA como um ‘hub’ de fluxo genético. Além disso, com base nas proporções dos isótopos estáveis de hidrogênio, existem fortes evidências de uma migração reversa (em direção ao sul) de *H. zea* do continente dos EUA para Porto Rico. Nosso estudo destaca as implicações para os programas de MIP e MRI, enfatizando a necessidade de estratégias abrangentes que considerem tanto os padrões de migração para o norte quanto para o sul.

## Introduction

The phenomenon of insect migration is connected with the fundamental framework of ecological processes, global biodiversity, and worldwide agriculture. Migration is a behavioral pattern observed in animals, involving travel between habitats in search of food, improved conditions, or for reproductive purposes. It typically involves a series of physiological, morphological, and behavioral adaptations (Johnson 1969, Dingle 2014a, Sappington 2024). Unlike other forms of animal movement, such as short-range dispersal, migration is characterized by long-distance movement, usually occurs seasonally, and often involves a return journey (Johnson 1969, Chapman et al. 2011, Dingle 2014b, Hu et al. 2016). For insects, migration can be defined as a long-range movement event that commonly happens soon after adult emergence, involving persistent and directional flight (Wolf et al. 1990, Westbrook 2008, Chapman et al. 2010). Understanding the pattern of long-range movement is critical to gaining insight into the ecology and behavior of migratory pest insects. This understanding can, in turn, offer valuable ecological information when designing programs for more effective, sustainable management of pests in agricultural systems.

Migratory insect pests play a significant role in agricultural systems, often causing substantial injury to crops and leading to significant yield losses (Deutsch et al. 2018). Studies describing their migratory patterns are essential for establishing efficient pest management strategies. Weather and wind patterns greatly influence insect pest migration, as demonstrated in studies that use remote sensing methods combined with synoptic meteorology, field monitoring, aerial sampling, and spore- and pollen-marked specimens (Wolf et al. 1986, 1990, Lingren et al. 1994, Westbrook et al. 1998, Westbrook 2008). Although these studies shed light on the migratory behavior of lepidopteran pests (Westbrook 2008, Tessnow et al. 2023), the geographic limitations of these studies pose challenges to comprehensive measurements and the predictability of large-scale movements. An attractive and innovative approach to overcome these challenges is the use of stable isotope techniques associated with standardized predictive geospatial modeling approaches, which can provide rapid, cost-effective, and precise solutions compared to other methods used to track insect migration patterns on a continental scale (Courtiol et al. 2019, Clem et al. 2023, Dittemore et al. 2023). Stable isotopes have long been used to identify geographically distinct animal populations, with variations in stable isotope ratios within food webs serving as natural markers that can be linked to origin (Vogel et al. 1990, Hobson 1999). In the last 3 decades, there has been a growing interest in stable hydrogen isotopes (e.g., Vogel et al. 1990, Wassenaar and Hobson 1998). This method is based on the correlation between hydrogen isotope ratios in rainfall and those found in plants, which are subsequently reflected in higher trophic-level consumers (Wassenaar and Hobson 1998). Unlike other extrinsic techniques (e.g., radars, pollen analysis, tags), stable-hydrogen isotope analysis offers broader applicability to small migratory species like insects, allowing the tracking of migratory species across large isotopic gradients. In Lepidoptera, wings are an appropriate tissue for the use of this type of marker. The tissue is formed during the pupal stage, and it reflects the isotopic composition of the meteoric water assimilated during larval feeding (Wassenaar and Hobson 2003, Rubenstein and Hobson 2004).

**Fig. 1.**
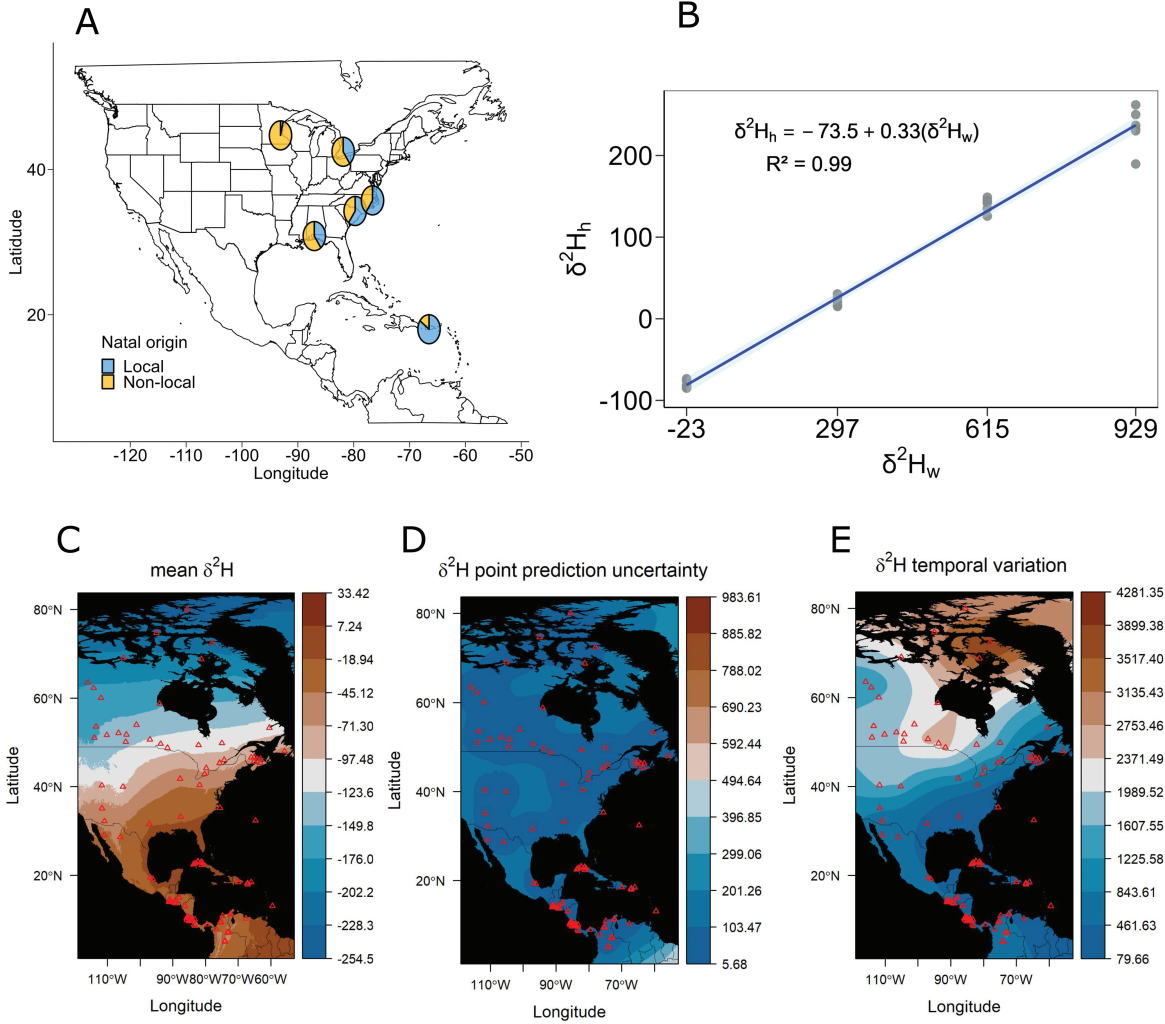
Continental isotopic gradient of δ^2^H. A) The proportion of estimated local and nonlocal individuals of *H. zea* based on hydrogen isotopic composition across a latitudinal gradient. B) Calibration equation linking dietary water and *H. zea* tissue δ^2^H in the laboratory experiment, which accounts for discrepancies in the source of δ^2^H (i.e., δ^2^H from water and δ^2^H from animal tissue). Dots indicate raw data: 6 per concentration of deuterated water (Supplementary Text). Isoscapes illustrating C) predicted mean δ^2^H value in space, D) the level of uncertainty in δ^2^H point predictions, which is used internally when performing assignments, and E) the residual temporal fluctuation reflecting intermonth variance in isotopic values over the years. Red triangles represent the locations of δ^2^H data collection (GNIP stations). δ^2^H values are represented in permil (‰).

We used stable hydrogen isotopic ratios (δ^2^H) in wing tissue as a biogeochemical marker to examine migratory patterns and estimate the native origins of *Helicoverpa zea* (Boddie) (Lepidoptera: Noctuidae) individuals collected across a wide latitudinal gradient in North America (Fig. 1A; Table 1; Supplementary Table S1). This species is recognized as one of the most destructive agricultural pests in the Americas, causing feeding injury and yield losses to major food, fiber, and oil crops, with a remarkable preference for reproductive tissues of vegetables, cotton (*Gossypium hirsutum* L.), soybean (*Glycine max* (L.) Merr.), and maize (*Zea mays* L.) (Harding 1976, Martin et al. 1976, Reay-Jones 2019, Hodgson et al. 2023). In tropical and subtropical regions, it is multivoltine, with a facultative diapause during the pupal stage (Hardwick 1965) triggered by temperature and photoperiod (Phillips and Newsom 1966, Clemmensen and Hahn 2015). Recent studies have modeled 3 overwintering survival zones for *H. zea* (Lawton et al. 2022), emphasizing its adaptability to different climatic conditions. Its migratory capacity enables the colonization of agricultural landscapes and the surge in population density in regions where it cannot survive the harsh winters (Slosser et al. 1975, Eger et al. 1983, Lawton et al. 2022). Finally, proposed long-distance migration is claimed as one of the major reasons for ineffective control of *H. zea* and increased practical resistance to pyrethroid insecticides, or toxins from *Bacillus thuringiensis* (Bt) Berliner expressed in transgenic crops (Westbrook et al. 1995, Jacobson et al. 2009, Jones et al. 2019, Dively et al. 2023). Therefore, *H. zea* serves as an exemplary model for studying migratory patterns of pest insects to advance our understanding of its ecology and improve Integrated Pest Management (IPM) and Insect Resistance Management (IRM) programs.

**Table 1.** Locations of sample collection, geographic position, month and year of collection, and number of *Helicoverpa zea* samples collected in the border of corn crops. Additional information in Supplementary Table S1

Sample locations	Latitude (N)	Longitude (W)	Month, year	Sample per month	Total
Ontario, Canada	42.45	−81.89	June/July/Oct 2022	4/5/1	10
Minnesota, USA	44.71	−93.11	September 2017	25	25
North Carolina, USA	35.76	−76.64	August 2022	5	5
South Carolina, USA	34.30	−79.74	August 2022	5	5
Florida, USA	30.78	−87.14	July/August 2022	4/1	5
Puerto Rico, USA	18.00	−66.51	February 2023	20	20

## Material and Methods

### Moth Collection

Moths were collected in Canada, the continental United States, and Puerto Rico (Table 1) using pheromone and light traps placed in corn crops. A total of 70 samples were collected and shipped (only wings from Puerto Rico) to the West Florida Research and Education Center in Jay, FL. The geographic positions, months of collection, and number of samples per location are listed in Table 1 (additional information in Supplementary Table S1). The right forewing was removed and placed in 2-ml Eppendorf tubes, which were then stored at −20 °C until the isotope assessment (see below).

### Isotopic Calibration

We established a calibration equation linking the amount-weighted mean annual δ^2^H in local precipitation (δ^2^H_p_), with δ^2^H in adult *H. zea* forewings (δ^2^H_h_). For that, we reared *H. zea* from egg to adult on multispecies lepidopteran diets using 4 types of water spiked with increasing concentrations of deuterated water (99%, Sigma-Aldrich product number 151882): (i) tap water (tw) alone, (ii) tw + 0.05 µl/l, (iii) tw + 0.10 µl/l, and (iv) tw + 0.15 µl/l. This approach allowed us to rear *H. zea* in a controlled environment, where they were fed a diet with a known isotopic value.

Larvae of *H. zea* were derived from a colony established from moths collected during the fall 2022 from non-Bt corn in Jay, FL. Insects were reared in a room under conditions of 25 ± 2 °C with 70% ± 10% RH and 14:10 (light: dark) photoperiod. When insects reached the pupal stage, they were placed in a vermiculite substrate and transferred to metal mating cages (23 cm diameter × 30 cm height), internally lined with paper towel as an oviposition substrate. Adults were fed with 10% honey solution, changed every 2 days (Rabelo et al. 2020). The second laboratory generation (F_2_) was used for the calibration experiment.

Eggs were collected and placed individually in 10-ml rearing cups with caps containing 4 ml of multispecies lepidopteran diet (Frontier Scientific Services Agriculture, product number F9772). The diet was prepared following manufacturer instructions: 930 ml of boiling water added to 162 g of diet powder + 3.3 ml of linseed oil and blended for 3–4 min. Insects were kept individually in the cups until moth emergence. After boiling, water samples were collected for analysis to represent its final condition as it was incorporated into the diet. Six moths from each treatment were used for isotope measurement (*n* = 24 in total).

### Isotope Assessment

For each sample (both laboratory-reared and wild-caught moths), the right forewing was removed using stainless-steel forceps and a paintbrush. We used 95% ethanol to clean the paintbrush and forceps between samples to prevent contamination and aid in the removal of remaining scales and any potential surface oil from the wings (Laroche et al. 2019, Jeong et al. 2021). After drying, the proximal half of the forewings was cut into very small pieces (ranging from 0.080 to 0.180 mg) and placed in 2-ml Eppendorf tubes and kept at −20 °C until isotope assessment.

A Thermo Electron DeltaV Plus isotope ratio mass spectrometer coupled with a ConFlo IV interface linked to a TCEA (high-temperature conversion elemental analyzer) was used. Samples and standards were weighed and loaded into 4 mm × 6 mm silver capsules and left in 96-well plates for 2 days. Exchangeable hydrogen could be exchanged equally for both samples and standards during this time, ensuring that the isotopic composition of the samples and standards remained comparable and consistent (Wassenaar and Hobson 2003). Two keratin standards (CBS—Caribou Hoof Environment Canada and KHS—Kudu Horn Environment Canada) were used to determine the stable hydrogen values of the nonexchangeable portion of the hydrogen (Wassenaar and Hobson 2003). Capsules were placed into a Zero Blank autosampler. Samples were placed in the oven at 1,400 °C, and the organic matter was thermally converted into gases. The hydrogen from the sample formed H_2_. The H_2_ was separated from other gases in a GC column at 90 °C. The column was a ceramic column with glassy carbon chips, a glassy carbon tube, a carbon ash collector, and a carbon funnel. The H_2_ gas was transferred with a helium carrier gas to a ConFlo IV interface and then passed to the IRMS where the isotopic analysis was done.

Hydrogen isotopic values (δ^2^H) were measured in the Stable Isotope Mass Spec Lab, University of Florida, Gainesville, FL, USA. A Picarro L2120-I isotopic liquid water and water vapor analyzer (Santa Clara, CA, USA) were used coupled with a Picarro A0211 high precision vaporizer and a CTC HTS PAL autosampler (Santa Clara, CA, USA). Precision was based on USGS42 = 2.99 ‰ (*N* = 11). Results were standardized based on 2 internal University of Florida water standards (UW Antarctic water and Lake Tulane water) that were calibrated using international standards (USGS49 and USGS50). All isotope results are reported in standard delta notation relative to Vienna Standard Mean Ocean Water.

### Isoscape Construction

*Helicoverpa zea* isoscapes in the American continent were computed using R 4.2.2 (R Core Team 2023) and the package Isorix 0.9.0 (Courtiol et al. 2019), a designed R package for a geographic isotopic assignment using mixed spatial models. We downloaded raw precipitation isotope hydrogen data (δ^2^H_p_) between 1961 and 2020 from the Global Network of Isotopes in Precipitation (GNIP) database (IAEA/WMO 2023). GNIP is an open-access database that is available upon user registration. We gathered information from weather stations located in different parts of Central and North America. Data were aggregated by year, weighted by precipitation amount, and then aggregated by location. Thus, each observation corresponded to the average and variance of isotope values collected in one location over time. This aggregation is necessary because precipitation rate can influence water molecules in soils, which has repercussions for food webs.

We fitted 2 interrelated geostatistical models using the function *isofit*(). The first was a mean model (a linear mixed-effects model) that described how amount-weighted average δ^2^H_p_ values vary geographically. In this model, the fixed effects were elevation and absolute value for latitude. The second model was a residual dispersal model (a gamma generalized linear mixed-effects model) that categorized δ^2^H_p_ variation at each location. In both models, 2 random effects were designated to account (i) for differential sample processing among data sites incorporating effects unrelated to a geographic location, such as microclimate and measurement error, and (ii) for Matérn autocorrelation to capture the spatial similarity in measurements close together.

These geostatistical models were then employed to construct an amount-weight precipitation isoscape to perform the geographic assignment of samples of unknown origins. We downloaded an elevation raster file from the Global Multiresolution Terrain Elevation Database 2010 and cropped it to include only the Central and North American area at approximate latitude ranges of 5°–80° and longitude ranges of −120° to −40°. A predicted isoscape was then generated based on the precipitation data, *H. zea* data, and the aggregated elevation raster using the function *isoscape*(). A calibration model (function *calibfit*(), method =  “lab”) characterizing the relationship between the isotopic composition of *H. zea* wing tissue and water samples was fitted using results from the isotopic calibration experiment.

### Natal Origin Assignment

The geographical assignment of the 70 *H. zea* samples was conducted using the *isofind*() function. This function executed an assignment test for each sample at every potential location using the predicted isoscape. For each candidate location, the isofind function assessed whether the isoscape value at an unknown sample’s local origin aligned with the predicted value at the candidate location. Consequently, a test statistic was calculated based on the difference between these 2 values. The similarity between the assigned sample and the predicted value on the isoscape was translated into probability values through 95% confidence intervals. Consequently, values approaching 1 indicated the most plausible origin of the unknown sample. We conducted this analysis for each location to identify potential migratory individuals. The classification of local and migratory *H. zea* was determined using the extract function to evaluate the probability of individuals originating from the capture location, as indicated by the isoscape. Individuals exhibiting a *P*-value ≤ 0.1 were indicative of a migratory status (Clem et al. 2023).

## Results

Our calibration isotopic curve revealed a strong correlation between the δ^2^H_h_ isotopic ratios in *H. zea* wings reared under controlled conditions and the dietary water source with δ^2^H_w_ (*r*^2^ = 0.99, Fig. 1B; Supplementary Table S2). The application of the 2 geostatistical models to quantify the prediction of the average δ^2^H value in space, its temporal variation, and its levels of uncertainty showed a continental isotopic gradient. δ^2^H values became more negative as latitude increased (Fig. 1C–E), highlighting the significant role of latitude in δ^2^H composition across the northern hemisphere.

**Fig. 2.**
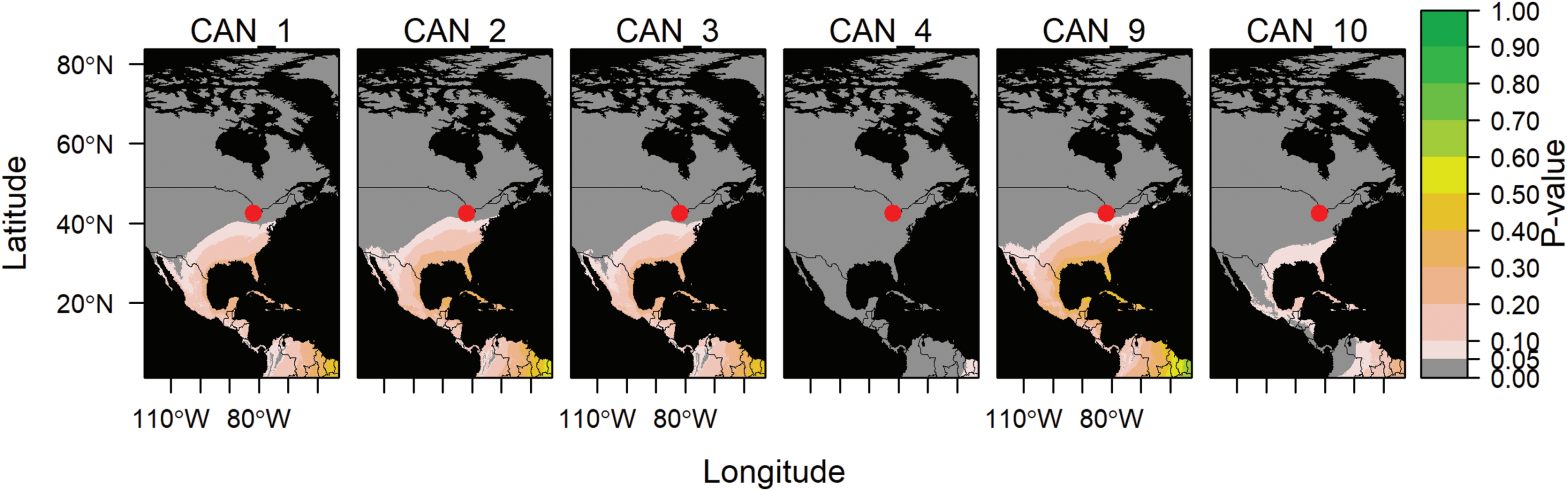
Northward migration of lower latitude populations of *Helicoverpa zea*. *Helicoverpa zea* specimens were collected in Canada with a nonlocal estimated origin. Each panel represents a single specimen, with the site of collection indicated by a red circle. Refer to Supplementary Table S1 for specimen ID details. *P*-values close to 1 (green) indicate the moth is highly likely to come from that region.

**Fig. 3.**
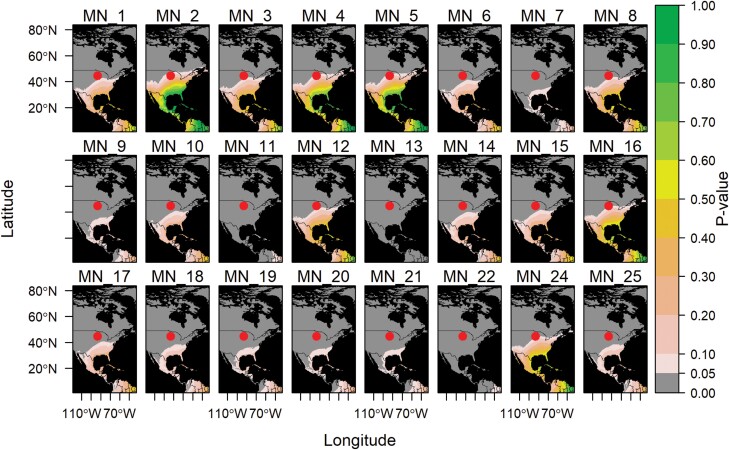
Northward migration of lower latitude populations of *Helicoverpa zea*. *Helicoverpa zea* specimens were collected in Minnesota with a nonlocal estimated origin. Each panel represents a single specimen, with the site of collection indicated by a red circle. Refer to Supplementary Table S1 for specimen ID details. *P*-values close to 1 (green) indicate the moth is highly likely to come from that region.

We determined the natal origin of our samples collected across the latitudinal gradient by performing geographic assignments for each sample at every potential origin using the predicted isoscape (Supplementary Figs. S1 and S2). Overall, the northward spring/summer migration of *H. zea* was clearly indicated using the biogeochemical marker δ^2^H. When assigning natal origins, we found that *H. zea* moths collected in Ontario, Canada, with nonlocal δ^2^H values (60% of the samples, Fig. 1A; Supplementary Fig. S3; Supplementary Table S1) had estimated points of migration origin from the Midwest United States extending to the southern and southeastern United States, Central America, and the Caribbean (Fig. 2; Supplementary Fig. S3). The estimated distances ranged from 200 (Midwest) to 3,500 (South Central America) km, with a corresponding probability of origin increasing further south (Fig. 2). In the same way, 96% of the specimens collected in Minnesota with nonlocal δ^2^H values (Figs. 1A and 3; Supplementary Fig. S4; Supplementary Table S1) indicated origins probably in the midwestern, southwestern, or southeastern United States, extending into Central America and the Caribbean (Fig. 3). The probability of origin increased southwards, with the highest probabilities estimated in the southwestern (~1,500 km) and southeastern United States (~2,000 km), or the Caribbean (~3,000 km) (Fig. 3).

**Fig. 4.**
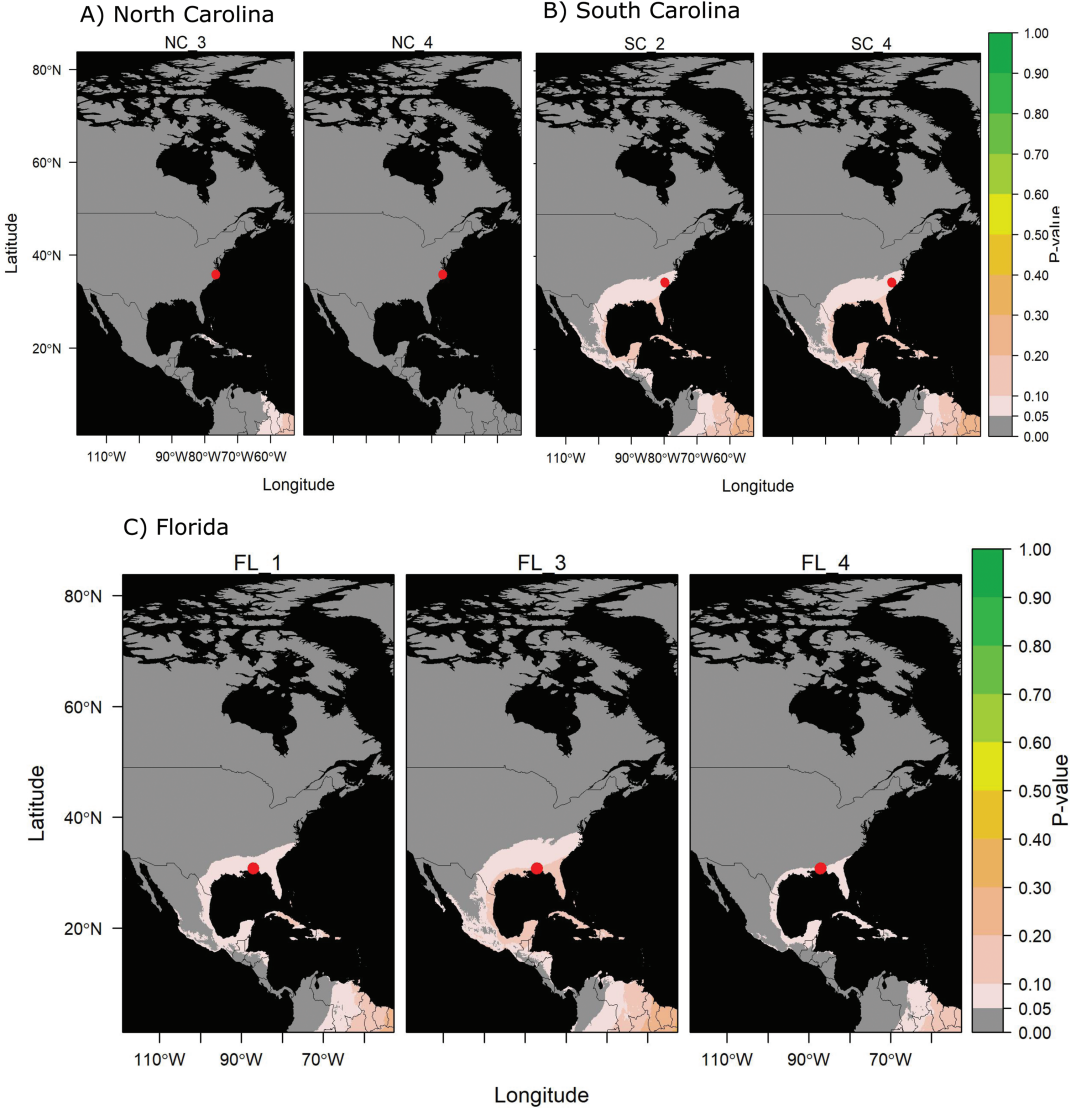
Blend of local and nonlocal assigned moths in Southeastern United States. *Helicoverpa zea* specimens collected in A) North Carolina, B) South Carolina, and C) Florida with nonlocal estimated origin. Each panel represents a single specimen, with the site of collection indicated by a red circle. Refer to Supplementary Table S1 for specimen ID details. *P*-values close to 1 (green) indicate the moth is highly likely to come from that region.

Moths of *H. zea* collected in North Carolina and South Carolina with nonlocal δ^2^H values (40% of the samples each, Fig. 4A and B; Supplementary Figs. S5 and S6; Supplementary Table S1) had higher probabilities of natal origin estimated from the southern United States, spanning from Texas to Florida, and the Caribbean (Fig. 4A and B). Specimens collected in Florida with nonlocal δ^2^H values (60% of the samples, Fig. 4C; Supplementary Fig. S7; Supplementary Table S1) had estimated probable origins in other parts of the southeastern United States, southwestern United States, and the Caribbean (Fig. 4C).

**Fig. 5.**
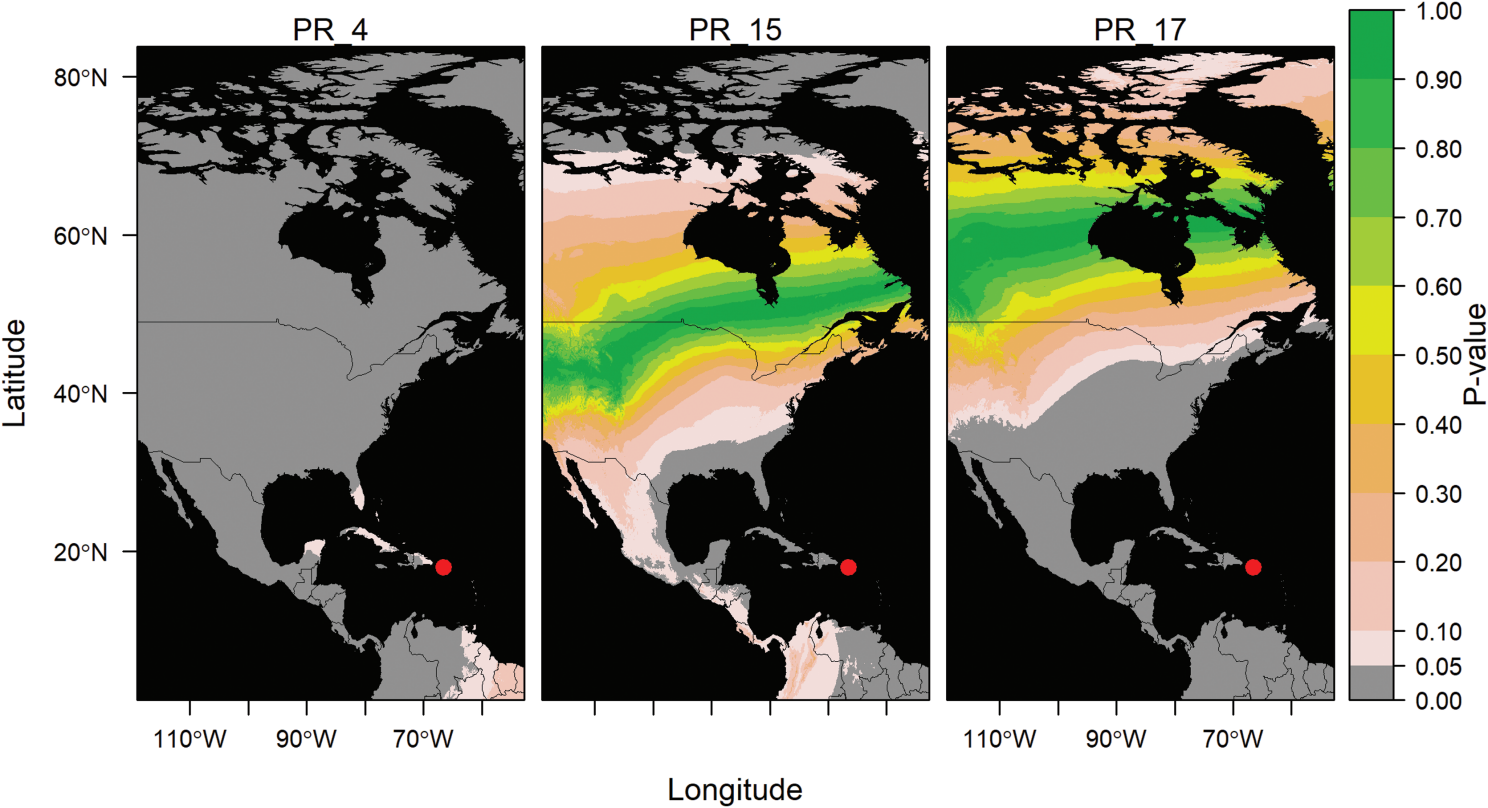
Indirect evidence of reverse migration. *Helicoverpa zea* specimens were collected in Puerto Rico with a nonlocal estimated origin. Each panel represents a single specimen, with the site of collection indicated by a red circle. Refer to Supplementary Table S1 for specimen ID details. *P*-values close to 1 (green) indicate the moth is highly likely to come from that region.

Our results also provide strong evidence of a reverse (southward) migration of *H. zea* from the continental United States to Puerto Rico. Three specimens collected in Puerto Rico with nonlocal δ^2^H values (15% of the samples, Figs. 1A and 5; Supplementary Fig. S8; Supplementary Table S1) had estimated origins in other parts of the Caribbean, southeastern and midwestern United States, and Canada (Fig. 5). The probability of origin increased northwards, with the lowest probabilities estimated at distances of at least 1,500 km and highest probabilities estimated around 4,000 km (Fig. 5).

## Discussion

Our findings validated the use of hydrogen isotopic ratios (δ^2^H) as biogeochemical markers, revealing potential large-scale latitudinal migratory patterns of *H. zea* in North America. This technology provides the advantage of linking the location of the collections with geographic origins and provides insight into the movement patterns of this species. Our findings on the migratory patterns of *H. zea* underscore the pivotal role of biogeochemical markers in refining management strategies, ensuring more effective and sustainable IPM programs in diverse agricultural landscapes. Below, we discuss our results in the context of each of the 3 latitudinal regions.

### Northward Migration of Lower Latitude Populations

Samples collected at higher latitudes (Ontario, Canada and Minnesota, USA) exhibited a greater proportion (60%–96%) of nonlocal individuals, with an increased probability of origin from the southeastern United States. These results support the models from Lawton et al. (2022), which proposed a revised overwintering boundary, as most samples from northern populations (Canada and Minnesota) classified as migratory had a high probability of originating below 35°N latitude (Figs. 2 and 3). In North America, climatic conditions are determinant for population dynamics of *H. zea* (Lawton et al. 2022), wherein populations tend to migrate northwards in a somewhat predictable manner (Westbrook 2008). For instance, it has been shown that *H. zea* populations undergo extensive dispersal each spring (Westbrook et al. 1997, Westbrook 2008, Westbrook and López 2010). With better climatic conditions and the beginning of the corn-growing season, *H. zea* migrates to more northern areas, engaging in windborne high-altitude flights (ranging from 60 to 1,768 m) with a 400-km displacement in 7.8 h (Westbrook 2008), which could result in a cumulative distance of 1,200 km per day. Moreover, during early to late autumn, *H. zea* from northern regions such as Canada and the northern United States is believed to migrate southward to areas with more favorable climates (Hardwick 1965, Gould et al. 2002, Sandstrom et al. 2007, Westbrook and López 2010, Lawton et al. 2022). Current evidence indicates that *H. zea* does not efficiently survive the winter in the northern regions, where it generally becomes a pest in the later part of the growing season in most years (Stinner et al. 1974, Morey et al. 2012).

Although most samples from northern populations exhibited a high likelihood of having a nonlocal origin, it is noteworthy that 4 of 10 samples (40%) in Canada and 1 of 25 samples (4%) in Minnesota indicated a local origin (Supplementary Figs. S3 and S4). These particular samples from Canada may suggest that some *H. zea* populations can overwinter above the 35th parallel. Traditionally, latitudes above the 40th parallel were considered to be beyond their typical overwintering range (Blanchard 1942, Hardwick 1965, Morey et al. 2012, Lawton et al. 2022). *Helicoverpa zea* can survive the winter at these marginal latitudes when local conditions, such as human-made structures that warm winter soil temperature, soil texture, and soil moisture, are optimized for pupal survival (Blanchard 1942). The probability of origin tends to increase as we move southward (Figs. 2 and 3), yet there remains a possibility that samples could originate from regions near the 35th parallel, which is about 280 km from the sampling location. Given potential similarities in isotope composition between these neighboring areas, these samples might reasonably be considered of local origin. Importantly, studies of this nature have the limitation of solely taking into account one biogeochemical marker (Hobson et al. 2021, Clem et al. 2023). However, advanced geostatistical approaches to assign the geographic origin of moths were used while controlling for spatial and temporal variance in rainfall (Courtiol et al. 2019). To enhance the precision of natal origin determination, future studies should combine other markers (Wassenaar and Hobson 1998, Hobson et al. 1999, Gould et al. 2002, Dittemore et al. 2023).

Additionally, the observation of a local origin for 1 of 25 samples in Minnesota offers insight into the migratory patterns and life cycle of *H. zea* in this region. One plausible reason for this local origin is the capture of an individual originating from the first generation of *H. zea*. In this region, there are typically 2 flight periods each summer. The first period, which starts around mid-June, is primarily comprised of moths migrating from the southern states. The second period, beginning in late July to early August, typically encompasses multiple flights and can contain migratory adult moths and F_1_ individuals from the first flight period (Hardwick 1965, Westbrook 2008, Burkness et al. 2009). Therefore, the sample, which was collected in September and categorized as having a local origin, could potentially represent an F_1_ individual from the first flight period. If climatic conditions continue to change in a way that allows for a greater number of early arriving migratory adults during the first flight period, this phenomenon, if left uncontrolled, could lead to an increase in the number of individuals, impacting population dynamics and crop outcomes. Hence, tracking migratory patterns linked to climatic conditions is of utmost importance for enhancing pest management programs, particularly in the case of *H. zea* (Beerwinkle et al. 1994, Lawton et al. 2022, Zhao et al. 2022).

### The Blend of Local and Nonlocal Assigned Moths in the Southeastern United States

We observed that *H. zea* samples from the southeastern United States (Florida, North Carolina, and South Carolina) were composed of a mix of local and nonlocal individuals (40%–60% migratory), showing a decrease in the proportion of migratory individuals compared to the northern populations. However, we did not find any samples originating from the northern region but from other regions in the southern United States. Samples in this region (Florida, North Carolina, and South Carolina) were collected in July and August (Table 1), which may be before moths in the northern regions begin to migrate southwards. By employing radar and meteorological data to assess the flight behavior of noctuid insects, Beerwinkel et al. (1994) observed that migration-like movements predominantly occurred from North to South during the autumn season. If *H. zea* adults from northern latitudes begin their migration at the end of summer and the beginning of fall, we can expect a greater number of individuals in the southern regions of the United States by late September.

Our findings suggest that there may be migratory or dispersal movements originating from other southern areas, including North Central America and the Caribbean. Lingren et al. (1994) observed that *H. zea* specimens captured in the US state of Oklahoma may have originated approximately 1,515 km away from the collection site, encompassing regions such as southern Florida, the Bahamas, Cuba, the Yucatan Peninsula, and northern Central America. This observation is consistent with our results, as samples from Florida, North Carolina, and South Carolina classified as nonlocal individuals showed estimated origin probabilities from the southeastern United States, North Central America, and the Caribbean (Fig. 4). Therefore, our observations suggest a mix of local and nonlocal *H. zea* individuals in southern regions like Florida, North Carolina, and South Carolina, with no specimens traced back from the north, potentially due to still favorable conditions in the north at the time of data collection. These findings underscore the significance of the southeastern United States as a crucial hub for genetic flow, facilitated by the influx of migrating moths into this region, which has also been proposed for another noctuid pest, *Spodoptera frugiperda* (Westbrook et al. 2019).

### Indirect Evidence of Reverse Migration

The documentation of potential reverse migration, with 15% of the *H. zea* moths in Puerto Rico exhibiting the isotopic composition of migrants during the crop season, suggests a significant capacity for long-range movement, including a portion of this journey occurring over the Gulf of Mexico. The study performed by Lingren et al. (1994), which involved specimens of *H. zea* marked with *Citrus* pollen, proposed that moths captured in Oklahoma carried markers that may have originated from the Caribbean region, implying that they had to cross the Gulf of Mexico. In the same way, several Noctuidae species have been documented flying over the Gulf of Mexico, with specimens found as far as 320 km from the nearest mainland (Wolf et al. 1986). The presence of ships, oil platforms, and floating *Sargassum* spp. in the Gulf of Mexico may provide suitable resting sites for these migrating moths (Wolf et al. 1986). Reverse migration of *H. zea* was also hypothesized based on studies of the carbon isotopic composition of moths trapped in the southern United States during the end of the crop season (Gould et al. 2002). In this study, a predominance of collected moths from larvae fed on plants with metabolic physiology C4 was detected in a landscape dominated by cotton and soybean (C3 plants). Therefore, the origin of the moths was attributed to a possible return of moths from the Midwest (Gould et al. 2002).

Reverse migration has previously been conjectured for Noctuid species based on entomological and weather radar observations associated with weather conditions (Farrow and Daly 1987, Beerwinkle et al. 1994). Based on the biogeochemical mark of hydrogen isotopic ratios (δ^2^H), *H. zea* moths have the capacity to cover long distances, traversing both mainland and sea to reach more favorable conditions for overwintering. These returning flights need to have supportive windborne conditions, such as the passage of cold fronts in autumn (Beerwinkle et al. 1994, Krauel et al. 2015). In the same way that northward migrations of *H. zea* should be considered when designing IRM programs, our results confirm the risk of resistance in *H. zea* to insecticides and Bt toxins may be carried back to the source populations during a reverse migration. The north–south reverse migration in the American continent clearly has adaptive value by allowing for enhanced survival of *H. zea* moths during warmer winter months and, thus, the conservation of resistance alleles for future growing seasons. Overall, the results of hydrogen isotopic ratios provide robust evidence of reverse migration in *H. zea* across North America, highlighting the complex dynamics within insect populations and the potential implications for the management of this economic pest.

### Implications for IPM/IRM Programs

The inferences of spatial origin of *H. zea* in North America in the present study provide an opportunity to implement a continental forecast for *H. zea* migratory populations in an area-wide pest management approach (Knipling 1979, Fleischer et al. 2007, Hutchison 2015, Lawton et al. 2022). This framework has the potential to determine local and regional units for early pest detection and estimation of the risk of annual outbreaks, promoting the adoption of IPM, all of which can alleviate the use of preventive chemical control. The knowledge and forecasting of the spatial and temporal origin of population sources of *H. zea* can also contribute IRM programs to estimate pest sources and risk of selection pressure for the evolution of resistance to management tools, such as chemical control (Hodgson et al. 2023). High rates of gene flow and panmixis due to migrant populations were previously indicated in *H. zea* (Perera et al. 2020). In North America, this species is predominantly managed with insecticides and transgenic Bt cotton and Bt corn. Local production of populations relative to IRM is important as well. For example, the prevalence of Bt corn, cotton, and non-Bt soybean in the prior year is correlated with Bt (Cry1Ac) bioassay screening results for *H. zea* populations in a 1 km radius (Arends et al. 2021). In addition, a greater presence of Bt corn in the current season was associated with reduced *H. zea* injury to corn kernels, hinting at a potential interplay between *H. zea* dilution and differential Bt resistance status (Arends et al. 2022). Our results support the risk of selection pressure for resistant alleles and spreading resistance in local and migratory populations. Therefore, our results confirm the hypothesis that the adoption of insecticides and Bt crops in southern regions of the United States impacts the performance of these management tools in northern regions of the United States and in the southern regions of Canada (Hutchison et al. 2007, Jacobson et al. 2009, Dively et al. 2023).

The existence of migrant moths returning to overwintering regions indicates that the adoption of management tools in northern regions is necessary to alleviate the economic impact of *H. zea* at local scales, as well as the risk imposed by the continuing selection for resistance to insecticides and Bt technology in the United States. Moreover, IRM programs should consider reverse (north to south) migration when modeling the risk of resistance and monitoring susceptibility in *H. zea* populations. In the same way, genomic studies to develop markers to track changes in allelic frequency for resistance to Bt toxins and insecticides should assess the possibility of introgression of genes from migrants of *H. zea.*

## Supplementary Material

Supplementary material is available at *Environmental Entomology* online.

nvae034_suppl_Supplementary_Materials

## Data Availability

All data are available in Supplementary Materials.
