# Supplementary material for: Continental-scale migration patterns and origin of Helicoverpa zea (Lepidoptera: Noctuidae) based on a biogeochemical marker
Source: Environ Entomol. 2024 Apr 18;53(3):487–97. doi: 10.1093/ee/nvae034 (PMC11170220; doi:10.1093/ee/nvae034)
Supplement: nvae034_suppl_Supplementary_Materials [file nvae034_suppl_supplementary_materials.docx]

**Continental-scale migration patterns and origin of *Helicoverpa zea*** **(Lepidoptera: Noctuidae) based on a biogeochemical marker**

Paula-Moraes, S.V.^1*^, Calixto, E.S.^1^, Santos, A.A.^1^, Reay-Jones, F.P.^2^, Reisig, D.D.^3^, Farhan, Y.^4^, Smith, J.^4^, Hutchison, W.D.^5^

* Corresponding author: Silvana Vieira de Paula-Moraes, Jay Admin Rm. 11, UF/IFAS West Florida Research and Education Center, 4253 Experiment Rd., Hwy. 182, Jay, FL 32565, 850-983-7101, [paula.moraes@ufl.edu](mailto:paula.moraes@ufl.edu)

**SUPPLEMENTARY**

**Table S1:** Raw δ^2^H data were obtained from wild-caught specimens in Canada, Minnesota, North Carolina, South Carolina, Florida, and Puerto Rico. Migratory status was determined based on extracted p-values from δ^2^H isoscapes rasters (Local individuals: p > 0.1, Migratory individuals: p < 0.1). Collection date: year-month-day format.

| **Country** | **Collection**  **Date** | **Sample_ID** | **Latitude** | **Longitude** | **Sample value** | **Local P-value** | **Migratory Status** |
| --- | --- | --- | --- | --- | --- | --- | --- |
| Canada | 2022-06-01 | CAN_1 | 42.451376 | -81.888829 | -60.03 | 0.0190 | Migratory |
| Canada | 2022-06-01 | CAN_2 | 42.451376 | -81.888829 | -61.87 | 0.0265 | Migratory |
| Canada | 2022-06-08 | CAN_3 | 42.451376 | -81.888829 | -60.42 | 0.0204 | Migratory |
| Canada | 2022-06-22 | CAN_4 | 42.451376 | -81.888829 | -42.51 | 0.0004 | Migratory |
| Canada | 2022-07-29 | CAN_5 | 42.451376 | -81.888829 | -87.87 | 0.6506 | Local |
| Canada | 2022-07-29 | CAN_6 | 42.451376 | -81.888829 | -79.76 | 0.3173 | Local |
| Canada | 2022-07-29 | CAN_7 | 42.451376 | -81.888829 | -74.19 | 0.1683 | Local |
| Canada | 2022-07-29 | CAN_8 | 42.451376 | -81.888829 | -80.87 | 0.3550 | Local |
| Canada | 2022-07-29 | CAN_9 | 42.451376 | -81.888829 | -64.24 | 0.0397 | Migratory |
| Canada | 2022-10-04 | CAN_10 | 42.451376 | -81.888829 | -53.35 | 0.0050 | Migratory |
| Minnesota | 2017-09-13 | MN_1 | 44.706971 | -93.106705 | -64.71 | 0.0147 | Migratory |
| Minnesota | 2017-09-13 | MN_2 | 44.706971 | -93.106705 | -76.48 | 0.0999 | Migratory |
| Minnesota | 2017-09-13 | MN_3 | 44.706971 | -93.106705 | -66.32 | 0.0198 | Migratory |
| Minnesota | 2017-09-13 | MN_4 | 44.706971 | -93.106705 | -72.89 | 0.0592 | Migratory |
| Minnesota | 2017-09-13 | MN_5 | 44.706971 | -93.106705 | -73.86 | 0.0685 | Migratory |
| Minnesota | 2017-09-13 | MN_6 | 44.706971 | -93.106705 | -61.76 | 0.0083 | Migratory |
| Minnesota | 2017-09-13 | MN_7 | 44.706971 | -93.106705 | -52.6 | 0.0011 | Migratory |
| Minnesota | 2017-09-13 | MN_8 | 44.706971 | -93.106705 | -67.05 | 0.0226 | Migratory |
| Minnesota | 2017-09-13 | MN_9 | 44.706971 | -93.106705 | -54.42 | 0.0017 | Migratory |
| Minnesota | 2017-09-13 | MN_10 | 44.706971 | -93.106705 | -60.79 | 0.0068 | Migratory |
| Minnesota | 2017-09-13 | MN_11 | 44.706971 | -93.106705 | -47.32 | 0.0003 | Migratory |
| Minnesota | 2017-09-13 | MN_12 | 44.706971 | -93.106705 | -66.69 | 0.0212 | Migratory |
| Minnesota | 2017-09-13 | MN_13 | 44.706971 | -93.106705 | -34.82 | 0.0000 | Migratory |
| Minnesota | 2017-09-13 | MN_14 | 44.706971 | -93.106705 | -59.7 | 0.0055 | Migratory |
| Minnesota | 2017-09-13 | MN_15 | 44.706971 | -93.106705 | -62.13 | 0.0090 | Migratory |
| Minnesota | 2017-09-13 | MN_16 | 44.706971 | -93.106705 | -69.86 | 0.0365 | Migratory |
| Minnesota | 2017-09-13 | MN_17 | 44.706971 | -93.106705 | -60.61 | 0.0066 | Migratory |
| Minnesota | 2017-09-13 | MN_18 | 44.706971 | -93.106705 | -57.15 | 0.0032 | Migratory |
| Minnesota | 2017-09-13 | MN_19 | 44.706971 | -93.106705 | -54.56 | 0.0018 | Migratory |
| Minnesota | 2017-09-13 | MN_20 | 44.706971 | -93.106705 | -53.81 | 0.0015 | Migratory |
| Minnesota | 2017-09-13 | MN_21 | 44.706971 | -93.106705 | -53.04 | 0.0012 | Migratory |
| Minnesota | 2017-09-13 | MN_22 | 44.706971 | -93.106705 | -45.38 | 0.0002 | Migratory |
| Minnesota | 2017-09-13 | MN_23 | 44.706971 | -93.106705 | -105.56 | 0.7761 | Local |
| Minnesota | 2017-09-13 | MN_24 | 44.706971 | -93.106705 | -69.37 | 0.0337 | Migratory |
| Minnesota | 2017-09-13 | MN_25 | 44.706971 | -93.106705 | -57.27 | 0.0032 | Migratory |
| North Carolina | 2022-08-09 | NC_1 | 35.752667 | -76.631794 | -57.68 | 0.1128 | Local |
| North Carolina | 2022-08-09 | NC_2 | 35.752667 | -76.631794 | -60.02 | 0.1539 | Local |
| North Carolina | 2022-08-09 | NC_3 | 35.757297 | -76.639717 | -46.99 | 0.0204 | Migratory |
| North Carolina | 2022-08-09 | NC_4 | 35.852203 | -76.649842 | -38.2 | 0.0034 | Migratory |
| North Carolina | 2022-08-09 | NC_5 | 35.852203 | -76.649842 | -69.42 | 0.4277 | Local |
| South Carolina | 2022-08-23 | SC_1 | 34.289370 | -79.741270 | -77.1 | 0.8242 | Local |
| South Carolina | 2022-08-23 | SC_2 | 34.289370 | -79.741270 | -53.9 | 0.0748 | Migratory |
| South Carolina | 2022-08-24 | SC_3 | 34.301560 | -79.736830 | -60.34 | 0.1782 | Local |
| South Carolina | 2022-08-25 | SC_4 | 34.306250 | -79.740850 | -54.08 | 0.0768 | Migratory |
| South Carolina | 2022-08-25 | SC_5 | 34.442910 | -79.799970 | -62.11 | 0.2165 | Local |
| Florida | 2022-07-08 | FL_1 | 30.834281 | -87.056997 | -51.9 | 0.0667 | Migratory |
| Florida | 2022-07-08 | FL_2 | 30.775850 | -87.138261 | -59.77 | 0.1929 | Local |
| Florida | 2022-07-08 | FL_3 | 30.783022 | -87.135183 | -54.65 | 0.0991 | Migratory |
| Florida | 2022-07-09 | FL_4 | 30.783022 | -87.135183 | -49.97 | 0.0492 | Migratory |
| Florida | 2022-08-23 | FL_5 | 30.776181 | -87.148872 | -56.12 | 0.1213 | Local |
| Puerto Rico | 2023-02-01 | PR_1 | 18.000313 | -66.513270 | -62.69 | 0.4444 | Local |
| Puerto Rico | 2023-02-01 | PR_2 | 18.000313 | -66.513270 | -67.05 | 0.6405 | Local |
| Puerto Rico | 2023-02-01 | PR_3 | 18.000313 | -66.513270 | -64.07 | 0.5026 | Local |
| Puerto Rico | 2023-02-01 | PR_4 | 18.000313 | -66.513270 | -47.76 | 0.0733 | Migratory |
| Puerto Rico | 2023-02-01 | PR_5 | 18.000313 | -66.513270 | -85.2 | 0.4466 | Local |
| Puerto Rico | 2023-02-01 | PR_6 | 18.000313 | -66.513270 | -68.95 | 0.7356 | Local |
| Puerto Rico | 2023-02-01 | PR_7 | 18.000313 | -66.513270 | -70.51 | 0.8169 | Local |
| Puerto Rico | 2023-02-01 | PR_8 | 18.000313 | -66.513270 | -79.87 | 0.6874 | Local |
| Puerto Rico | 2023-02-01 | PR_9 | 18.000313 | -66.513270 | -67.3 | 0.6527 | Local |
| Puerto Rico | 2023-02-01 | PR_10 | 18.000313 | -66.513270 | -74.43 | 0.9724 | Local |
| Puerto Rico | 2023-02-01 | PR_11 | 18.000313 | -66.513270 | -73.78 | 0.9924 | Local |
| Puerto Rico | 2023-02-01 | PR_12 | 18.000313 | -66.513270 | -91.26 | 0.2432 | Local |
| Puerto Rico | 2023-02-01 | PR_13 | 18.000313 | -66.513270 | -71.97 | 0.8947 | Local |
| Puerto Rico | 2023-02-01 | PR_14 | 18.000313 | -66.513270 | -90.58 | 0.2620 | Local |
| Puerto Rico | 2023-02-01 | PR_15 | 18.000313 | -66.513270 | -111.31 | 0.0127 | Migratory |
| Puerto Rico | 2023-02-01 | PR_16 | 18.000313 | -66.513270 | -76.05 | 0.8853 | Local |
| Puerto Rico | 2023-02-01 | PR_17 | 18.000313 | -66.513270 | -127.56 | 0.0004 | Migratory |
| Puerto Rico | 2023-02-01 | PR_18 | 18.000313 | -66.513270 | -84.35 | 0.4814 | Local |
| Puerto Rico | 2023-02-01 | PR_19 | 18.000313 | -66.513270 | -58.55 | 0.2945 | Local |
| Puerto Rico | 2023-02-01 | PR_20 | 18.000313 | -66.513270 | -59.26 | 0.3175 | Local |

**Table S2:** Raw data from δ^2^H calibration experiment using lab method. Refer to methods for more details.

| **Species** | **δ^2^H exp treatment** | **δ^2^H_h_** |
| --- | --- | --- |
| Sample 1 | Tap water | -84.82 |
| Sample 2 | Tap water | -84.99 |
| Sample 3 | Tap water | -81.48 |
| Sample 4 | Tap water | -73.55 |
| Sample 5 | Tap water | -76.09 |
| Sample 6 | Tap water | -82.5 |
| Sample 7 | Tap water + 0.05 | 16.18 |
| Sample 8 | Tap water + 0.05 | 17.59 |
| Sample 9 | Tap water + 0.05 | 21.29 |
| Sample 10 | Tap water + 0.05 | 30.41 |
| Sample 11 | Tap water + 0.05 | 15.01 |
| Sample 12 | Tap water + 0.05 | 25.7 |
| Sample 13 | Tap water + 0.10 | 142.3 |
| Sample 14 | Tap water + 0.10 | 125.72 |
| Sample 15 | Tap water + 0.10 | 135.16 |
| Sample 16 | Tap water + 0.10 | 145.37 |
| Sample 17 | Tap water + 0.10 | 142.05 |
| Sample 18 | Tap water + 0.10 | 148.95 |
| Sample 19 | Tap water + 0.15 | 230.18 |
| Sample 20 | Tap water + 0.15 | 236.95 |
| Sample 21 | Tap water + 0.15 | 231.85 |
| Sample 22 | Tap water + 0.15 | 250.11 |
| Sample 23 | Tap water + 0.15 | 189.63 |
| Sample 24 | Tap water + 0.15 | 262 |


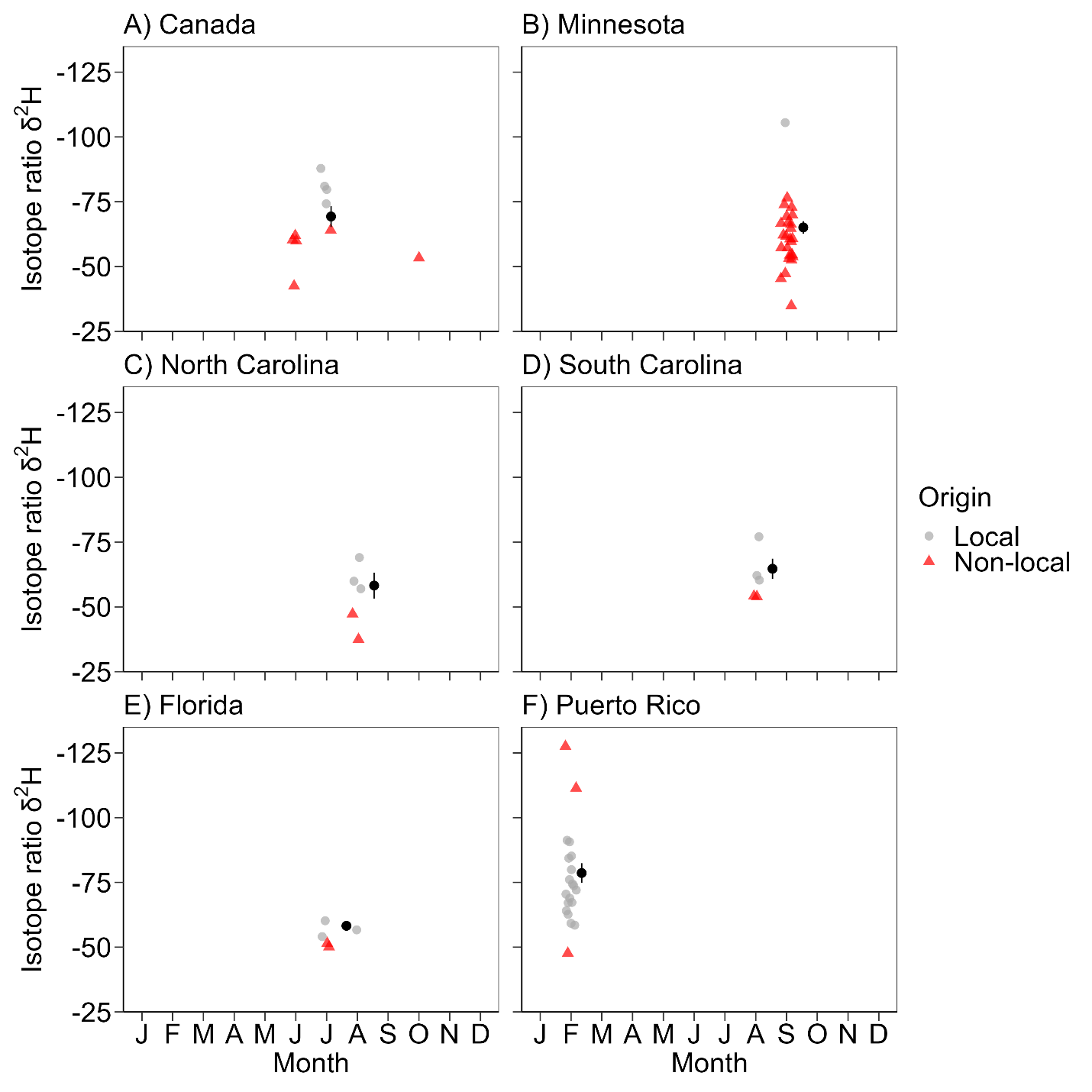


**Fig. S1 –** Raw δ^2^H values of *Helicoverpa zea* specimens captured in different parts of North America and the Caribbean. Black dots and lines represent the mean and standard error. Grey circles represent specimens highly likely to be of local origin (p-value > 0.1), while red triangles represent specimens highly unlikely to be of local origin (p-value < 0.1). Black dots with errors represent mean and standard error, respectively. Results are detailed in Table S1.


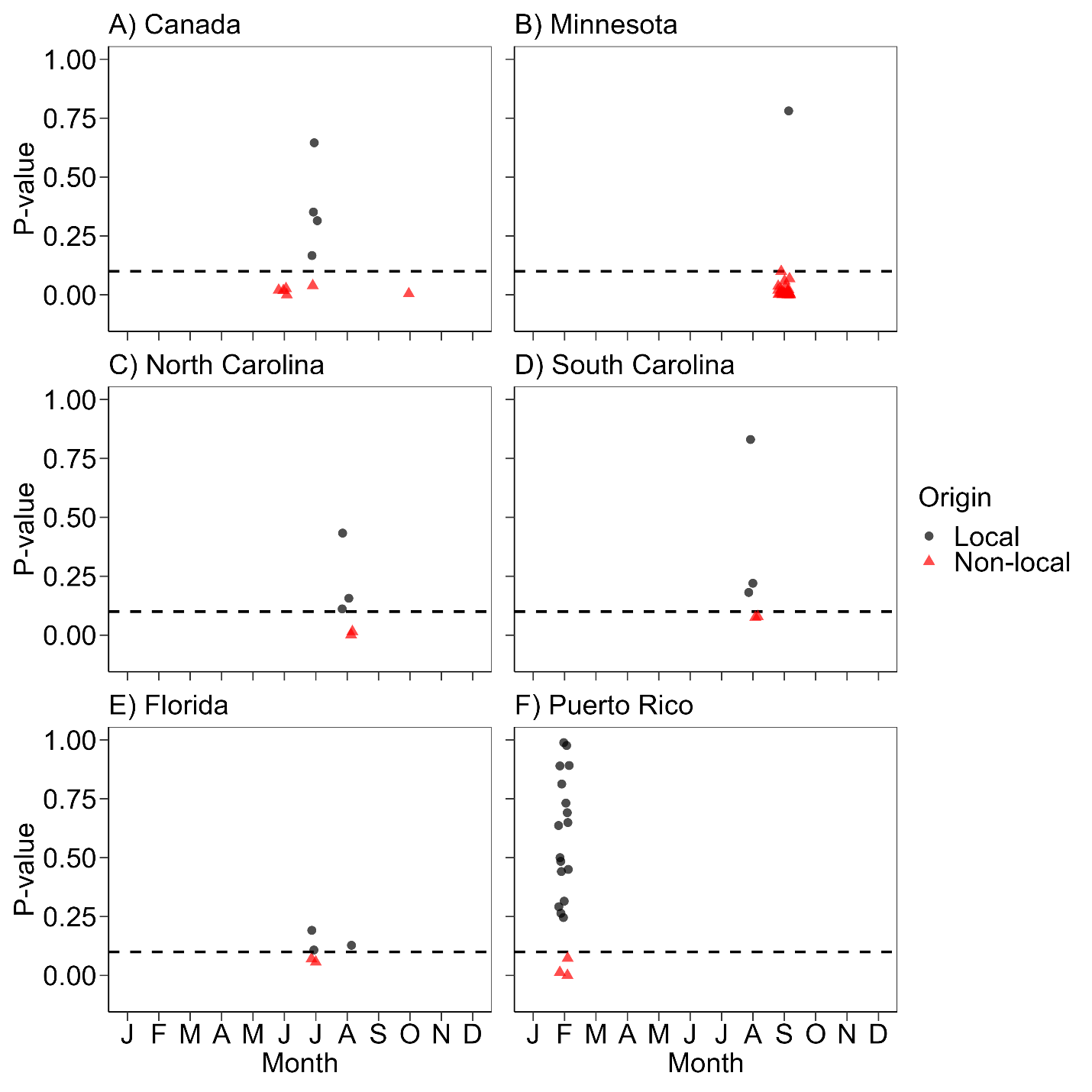


**Fig. S2 –** Likelihood of a specimen originating locally based on δ^2^H_h_ isoscape rasters. Grey circles represent specimens highly likely to be of local origin (p-value > 0.1), while red triangles represent specimens highly unlikely to be of local origin (p-value < 0.1). Results are detailed in Table S1. Dashed line indicates a p-value of 0.1


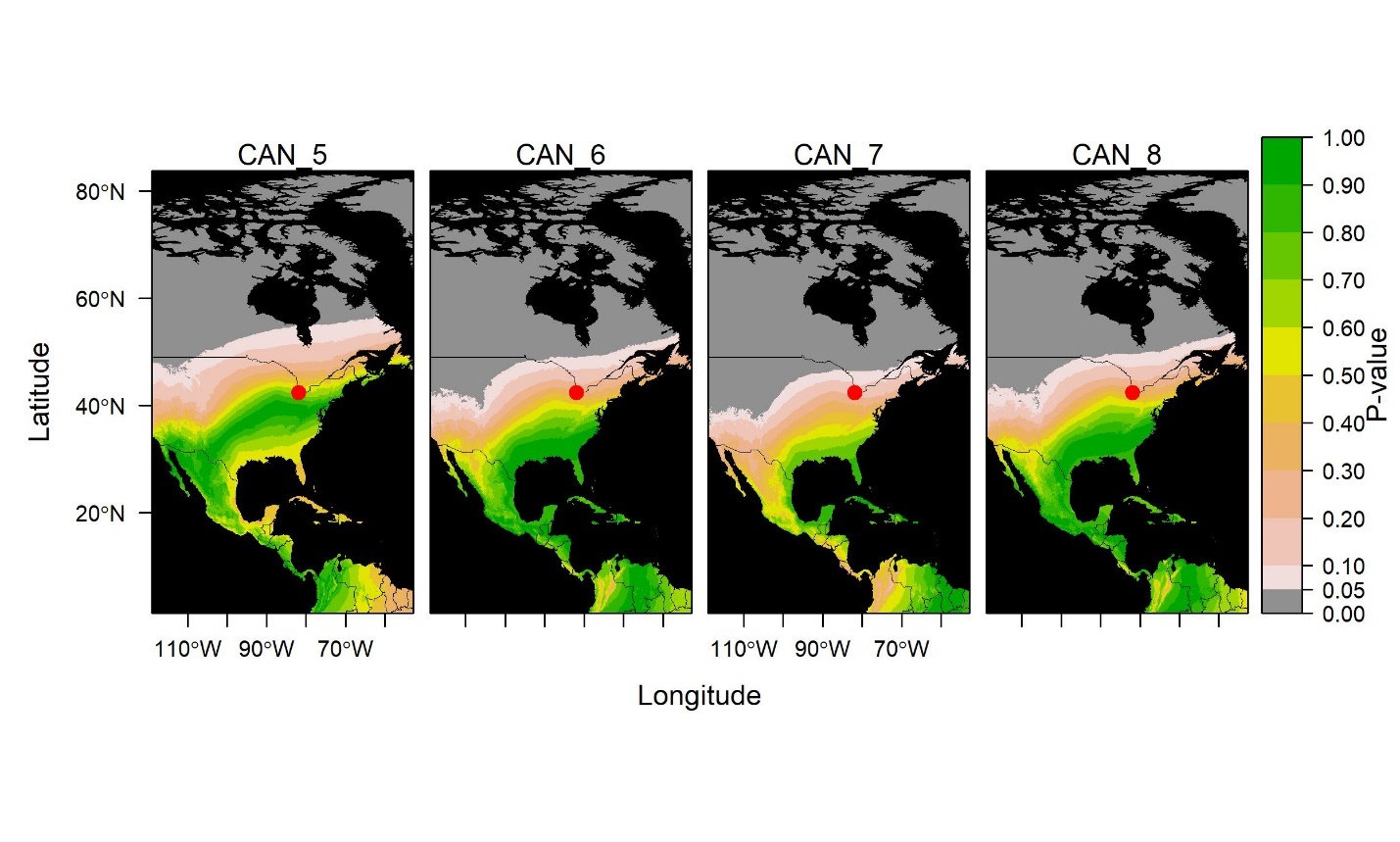


**Fig. S3 –***Helicoverpa zea* specimens collected in Canada with local estimated origin. Each panel represents a single specimen, with the site of collection indicated by a red circle. Refer to Table S1 for specimen ID details. δ^2^H values falling within the range of 10% to 100% probability of local origin (40% of the samples) were predominantly from mid-eastern U.S. to southern North America and Central America. P-values close to 1 (green) indicate the moth is highly likely to come from that region.


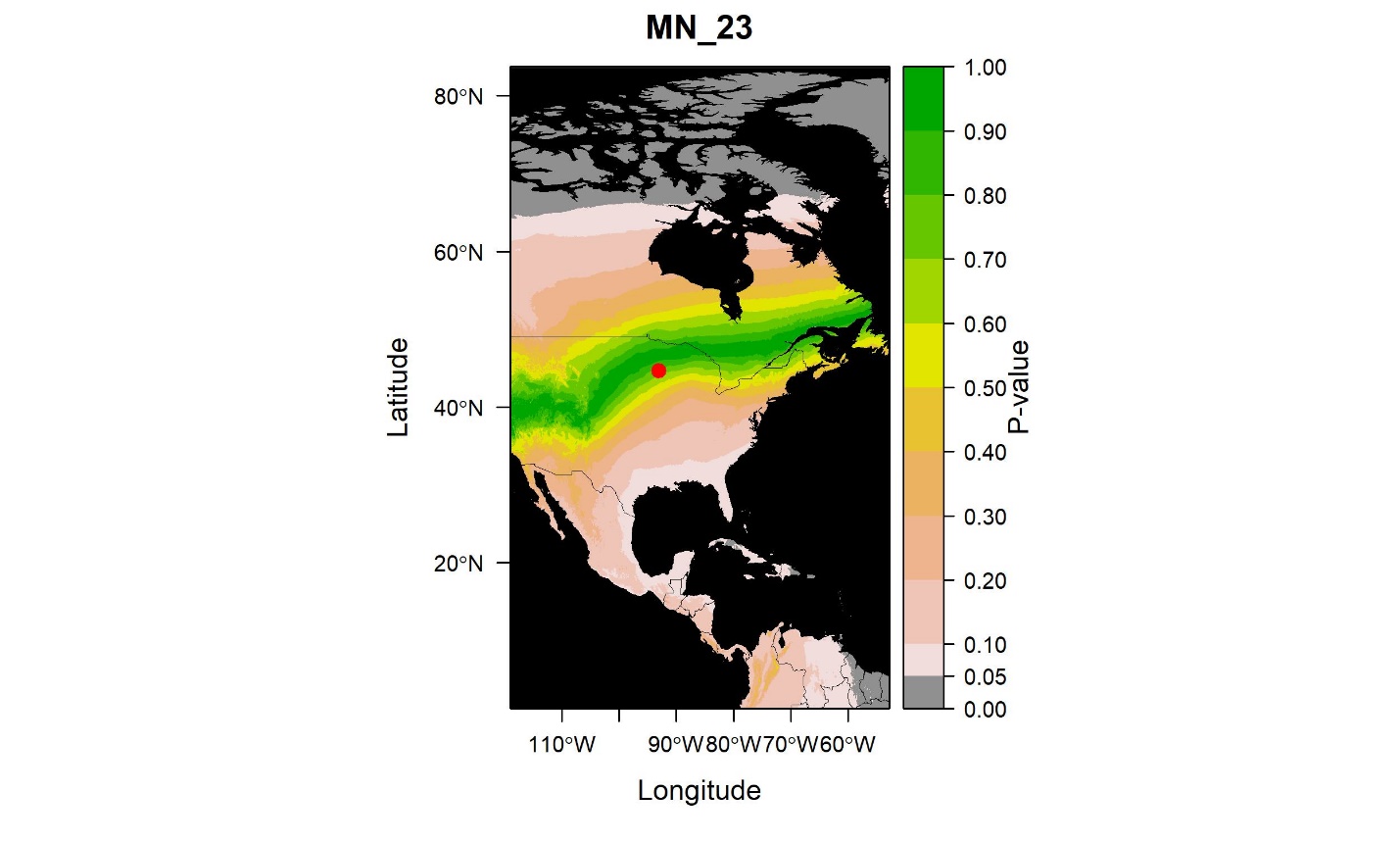


**Fig. S4 –***Helicoverpa zea* specimen collected in Minnesota with local estimated origin. The site of the collection is indicated by a red circle. Refer to Table S1 for specimen ID details. δ^2^H values falling within the range of 10% to 100% probability of local origin (4% of the samples) extended from the Midwest U.S. to Canada. P-values close to 1 (green) indicate the moth is highly likely to come from that region.


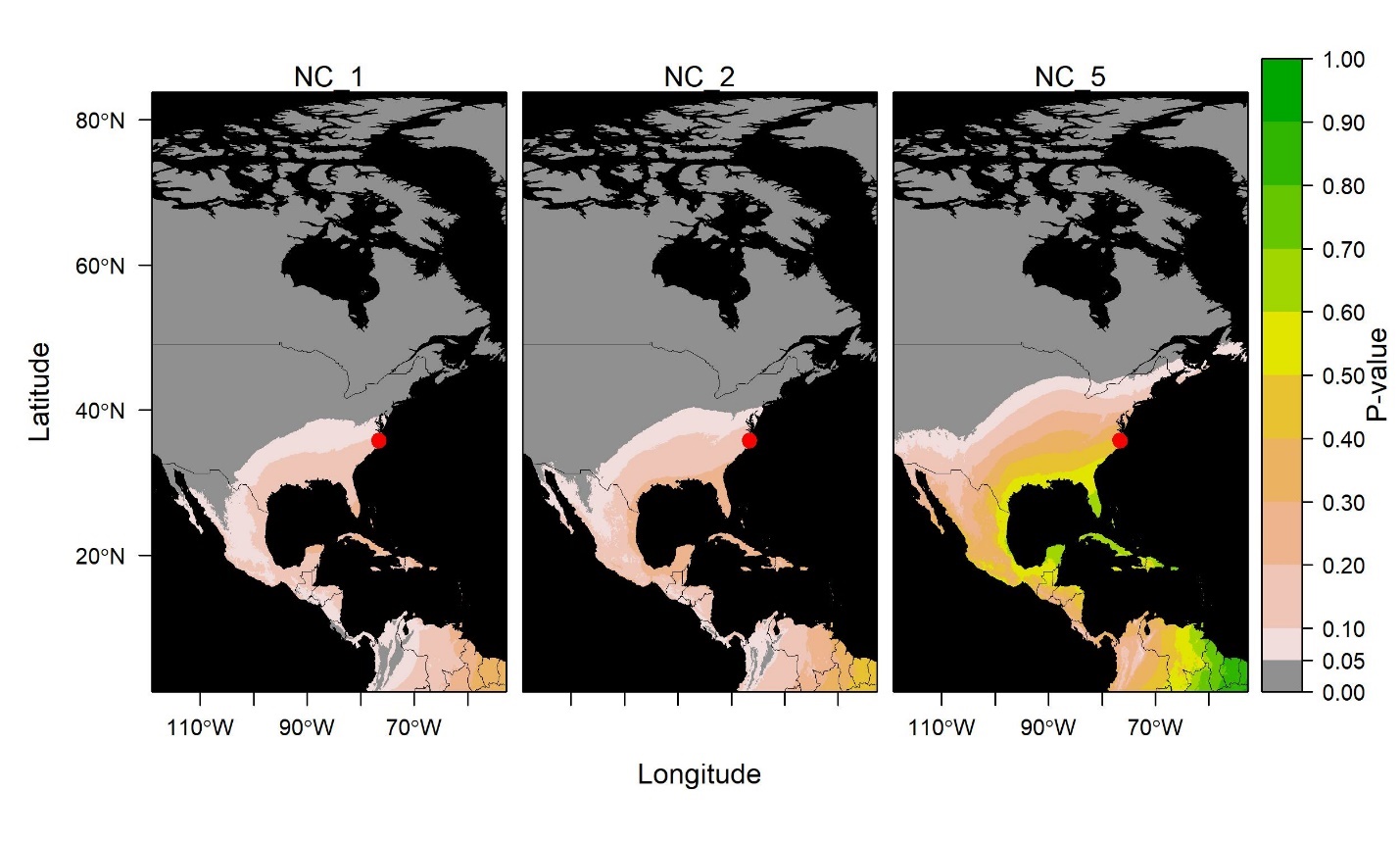


**Fig. S5 –***Helicoverpa zea* specimens collected in North Carolina with local estimated origin. Each panel represents a single specimen, with the site of collection indicated by a red circle. Refer to Table S1 for specimen ID details. δ^2^H values falling within the range of 10% to 100% probability of local origin (60% of the samples) were most likely from the local region, extending to the southern U.S. P-values close to 1 (green) indicate the moth is highly likely to come from that region.


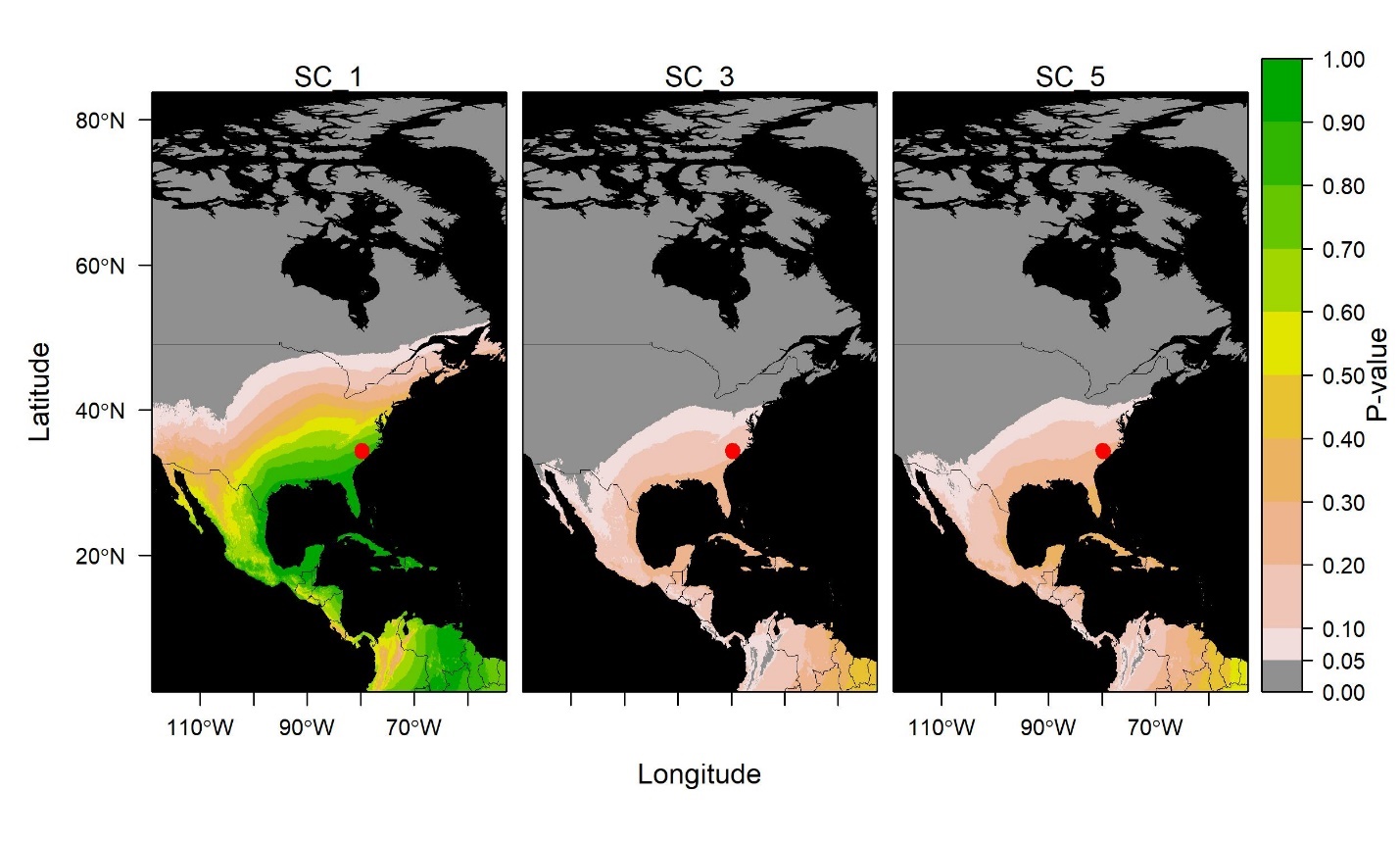


**Fig. S6 –** *Helicoverpa zea* specimens collected in South Carolina with local estimated origin. Each panel represents a single specimen, with the site of collection indicated by a red circle. Refer to Table S1 for specimen ID details. δ^2^H values falling within the range of 10% to 100% probability of local origin (60% of the samples) were most likely from the local region, extending to the southern U.S. P-values close to 1 (green) indicate the moth is highly likely to come from that region.


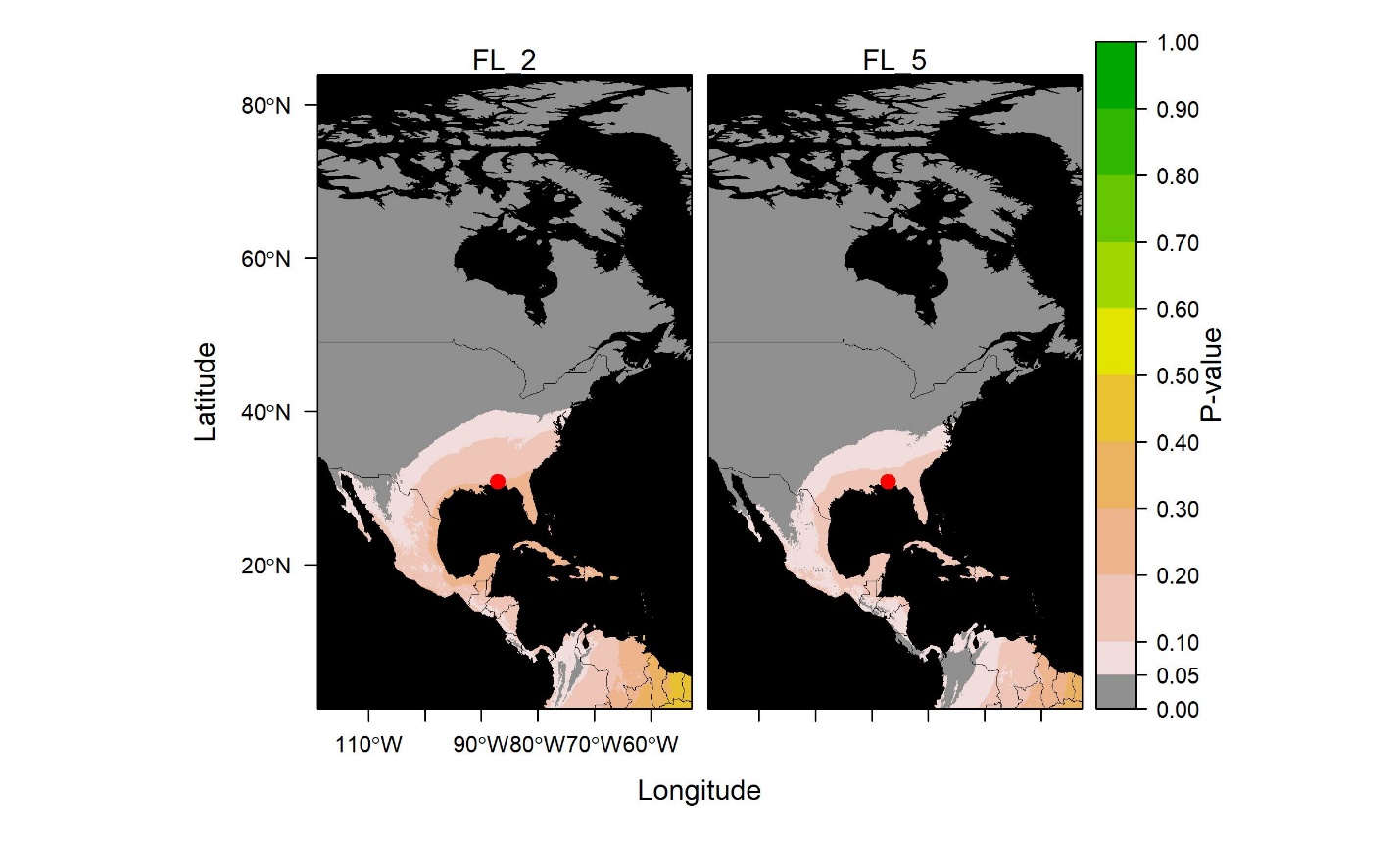


**Fig. S7 –** *Helicoverpa zea* specimens collected in Florida with local estimated origin. Each panel represents a single specimen, with the site of collection indicated by a red circle. Refer to Table S1 for specimen ID details. δ^2^H values falling within the range of 10% to 100% probability of local origin (40% of the samples) were most likely from the local region, extending to the southern U.S. P-values close to 1 (green) indicate the moth is highly likely to come from that region.

**
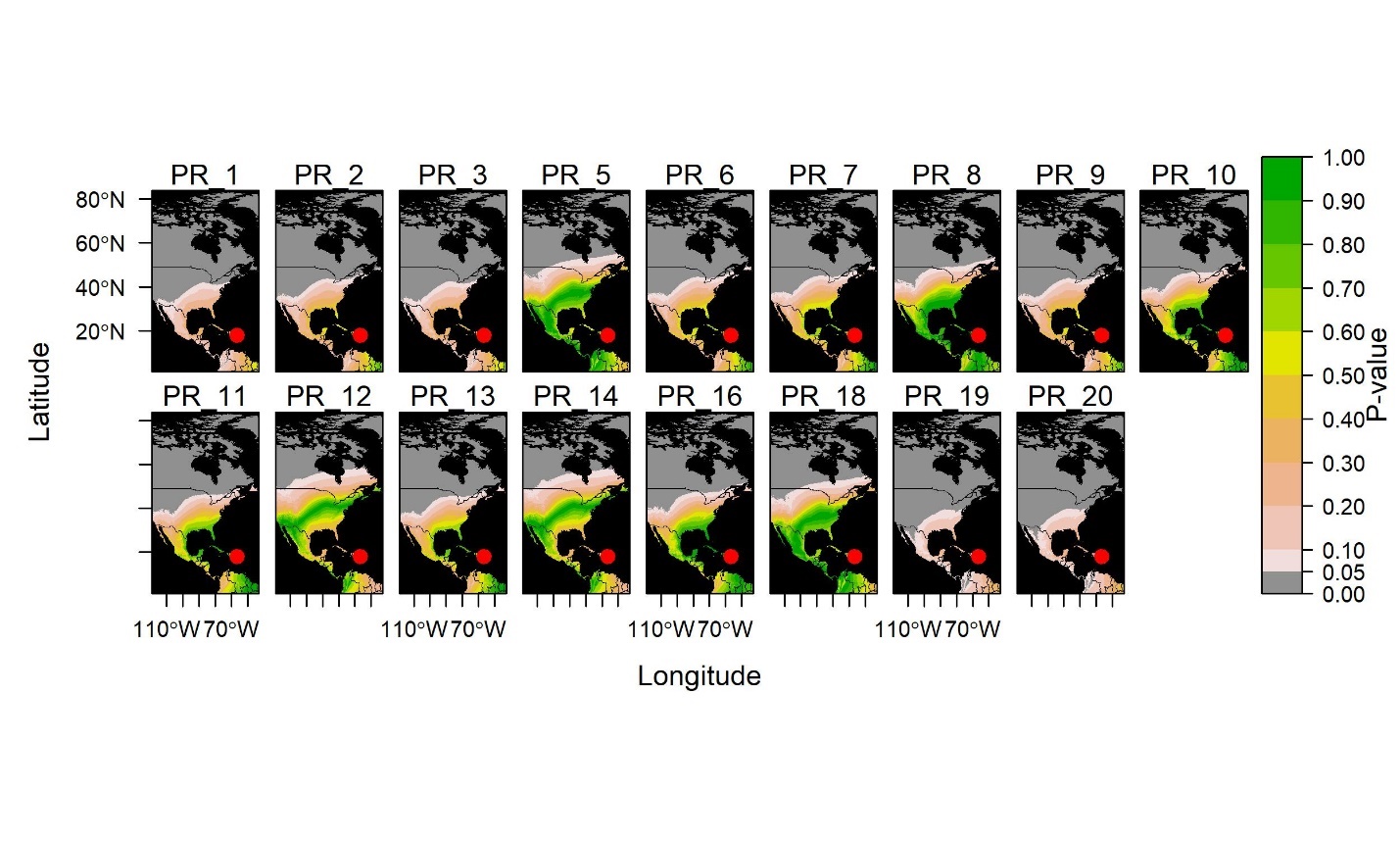
**

**Fig. S8 –** *Helicoverpa zea* specimens collected in Puerto Rico with local estimated origin. Each panel represents a single specimen, with the site of collection indicated by a red circle. Refer to Table S1 for specimen ID details. δ^2^H values falling within the range of 10% to 100% probability of local origin (85% of the samples) were most likely from the local region, extending to the southern U.S. P-values close to 1 (green) indicate the moth is highly likely to come from that region.
